# 869. Oral Encochleated Amphotericin B for Cryptococcal Meningitis: a Phase II Randomized Trial

**DOI:** 10.1093/ofid/ofac492.062

**Published:** 2022-12-15

**Authors:** Mucunguzi Atukunda, Enock Kagimu, Morris K Rutakingirwa, Lillian Tugume, Laura Nsangi, Abdu Musubire, Jane Gakuru, Timothy Mugabi, Andrew Akampurira, Kenneth Ssebambulidde, John Kasibante, Jayne Ellis, Edward Mpoza, Darlisha A Williams, Ann M Fieberg, Caleb Skipper, Mahsa Abassi, Kathy Huppler Hullsiek, David Meya, David R Boulware

**Affiliations:** Infectious Diseases Institute, Makerere University, Kampala, Kampala, Uganda; Infectious Diseases Institute, Makerere University, Kampala, Kampala, Uganda; Infectious diseases institute, College of health sciences, Makerere University, Kampala Uganda, Kampala, Kampala, Uganda; Infectious Diseases Institute, Makerere University, Kampala, Kampala, Uganda; Infectious Diseases Institute, Makerere University, Kampala, Kampala, Uganda; Infectious Diseases Institute, Makerere University, Kampala, Kampala, Uganda; Infectious Diseases Institute, Makerere University, Kampala, Kampala, Uganda; Infectious Diseases Institute, Makerere University, Kampala, Kampala, Uganda; Infectious Diseases Institute, Makerere University, Kampala, Kampala, Uganda; Infectious Diseases Institute, Makerere University, Kampala, Kampala, Uganda; Infectious Diseases Institute, Makerere University, Kampala, Kampala, Uganda; Infectious Diseases Institute, Makerere University, Kampala, Kampala, Uganda; Infectious Diseases Institute, Makerere University, Kampala, Kampala, Uganda; University of Minnesota, Minneapolis, Minnesota; University of Minnesota, Minneapolis, Minnesota; University of Minnesota, Minneapolis, Minnesota; University of Minnesota, Minneapolis, Minnesota; University of Minnesota, Minneapolis, Minnesota; Infectious Diseases Institute, Makerere University, Kampala, Kampala, Uganda; University of Minnesota, Minneapolis, Minnesota

## Abstract

**Background:**

Intravenous (IV) amphotericin B is the gold standard treatment of severe mycoses. A new orally absorbed, less-toxic formulation of amphotericin has been developed (Matinas Biopharma). We evaluated the efficacy of this novel anti-fungal agent amongst adults with cryptococcal meningitis.

**Methods:**

We conducted a phase II randomized clinical trial testing oral encochleated amphotericin B (cAMB) versus IV amphotericin B for first episode cryptococcal meningitis in Kampala, Uganda from December 2020 to August 2021. Participants were HIV-positive, CSF cryptococcal antigen (CrAg) positive, and had the capacity to consent and take oral medications (GCS=15). Participants in the experimental arm received two loading doses of either IV deoxycholate amphotericin B 1.0 mg/kg/day or liposomal amphotericin 3 mg/kg/day, followed by 1.8g oral cAMB daily in 6 divided doses through 2 weeks with flucytosine (5FC) at 100mg/kg/day, and thereafter cAMB at 1.2g daily in 4 divided doses through 6 weeks. Participants in the control arm received 7 days of IV amphotericin B (deoxycholate or liposomal) with 5FC, then 7 days of fluconazole 1200mg/day. After 14 days, all participants received fluconazole 800mg/day through 10 weeks and thereafter a maintenance dose of 200mg/day.

**Results:**

We randomized 40 participants to oral cAMB + 5FC and 30 control participants to IV amphotericin + 5FC. With cAMB the 30-day survival was 97.5% (39/40) and 18-week survival was 90% (36/40) compared with 87% (26/30) 18-week survival in IV amphotericin controls.

The CSF Early Fungicidal Activity (EFA) was lower with oral cAMB (mean EFA = 0.42 log_10_*Cryptococcus* CFU/mL/day; 95%CI, 0.29 to 0.54) versus IV amphotericin (mean EFA = 0.52 log_10_ CFU/mL/day; 95%CI, 0.39 to 0.66). Among those CSF culture positive at baseline, CSF sterility was achieved by 2 weeks in 65% (24/37) of cAMB participants and 68% (17/25) of controls.

Grade >=3 laboratory adverse events were more common with IV amphotericin. Grade 3–4 anaemia occurred in 10% (n=4) with cAMB versus 37% (n=11) with IV amphotericin. Grade 3 hypokalaemia (< 3mEq/L) occurred in 5% (n=2) with cAMB versus 27% (n=8) with IV amphotericin.

**Conclusion:**

Novel oral cAMB appears to be a safe agent with promising efficacy for HIV-related cryptococcal meningitis.

**Disclosures:**

**All Authors**: No reported disclosures.

